# Good Manufacturing Practices and Microbial Contamination Sources in Orange Fleshed Sweet Potato Puree Processing Plant in Kenya

**DOI:** 10.1155/2018/4093161

**Published:** 2018-04-02

**Authors:** Derick Nyabera Malavi, Tawanda Muzhingi, George Ooko Abong'

**Affiliations:** ^1^Department of Food Science, Nutrition and Technology, University of Nairobi, P.O. Box 29053, Nairobi 00625, Kenya; ^2^International Potato Centre (CIP), Sub-Saharan Africa (SSA) Regional Office, Old Naivasha Road, P.O. Box 25171, Nairobi 00603, Kenya

## Abstract

Limited information exists on the status of hygiene and probable sources of microbial contamination in Orange Fleshed Sweet Potato (OFSP) puree processing. The current study is aimed at determining the level of compliance to Good Manufacturing Practices (GMPs), hygiene, and microbial quality in OFSP puree processing plant in Kenya. Intensive observation and interviews using a structured GMPs checklist, environmental sampling, and microbial analysis by standard microbiological methods were used in data collection. The results indicated low level of compliance to GMPs with an overall compliance score of 58%. Microbial counts on food equipment surfaces, installations, and personnel hands and in packaged OFSP puree were above the recommended microbial safety and quality legal limits. Steaming significantly (*P* < 0.05) reduced microbial load in OFSP cooked roots but the counts significantly (*P* < 0.05) increased in the puree due to postprocessing contamination. Total counts, yeasts and molds, Enterobacteriaceae, total coliforms, and* E. coli *and* S. aureus* counts in OFSP puree were 8.0, 4.0, 6.6, 5.8, 4.8, and 5.9 log_10_ cfu/g, respectively. In conclusion, equipment surfaces, personnel hands, and processing water were major sources of contamination in OFSP puree processing and handling. Plant hygiene inspection, environmental monitoring, and food safety trainings are recommended to improve hygiene, microbial quality, and safety of OFSP puree.

## 1. Introduction

Sweet potato is one of the most important food crops in Kenya. According to FAOSTAT [1], sweet potato production in Kenya stood at 697,364 tonnes in the year 2016. Kenya is the sixth largest producer of sweet potato in Africa with an average yield of 8.2 tonnes/ha [2]. In a review by Abong' et al. [3], major sweet potato producing counties in Kenya as per the year 2014 were Homa Bay, Busia, Migori, and Bungoma counties. Many cultivars of sweet potatoes differentiated by color and shape are cultivated in Kenya. The flesh color ranges from white, cream, purple, yellow, and orange [4]. Breeding and utilization of biofortified Orange Fleshed Sweet Potato (OFSP) variety is being promoted in Kenya and other sub-Saharan (SSA) countries by research organizations due its high beta-carotene (provitamin A) content [5–8]. OFSP is an important food crop for income generation, addressing vitamin A deficiency, and food insecurity in SSA [6, 9]. Depending on the region, OFSP cultivars grown in Kenya include Kabode, Vitaa, SPK 004, and Ejumula [10]. Sweet potato is often processed into purees that are subsequently incorporated as a food ingredient in baby foods, puddings, doughnuts, buns, breads, cakes, cookies, soups, and beverages [6, 11]. Since the year 2015, the International Potato Centre (CIP) has been working with a privately owned OFSP puree processing company operated on a small-scale basis and one of the largest retail chain stores in Kenya to promote utilization of OFSP puree in bakery applications and enhance intake of vitamin A among the urban population [12].

Most studies are currently focusing on nutritional and socioeconomic benefits of OFSP but little effort has been directed towards enhancing food safety along the OFSP puree value chain that has gained prominence in Kenya. Food safety problems are more pronounced in developing countries where food production is frequently done under unsanitary conditions [13]. Microbial quality and safety of foods can constantly be achieved by implementation and adherence to Good Hygiene and Good Manufacturing Practices (GMPs) in processing. There is a potential of contamination in sweet potato puree along the process line that could result from poor hygiene practices and contamination from the processing environment [11]. Microbial food contamination is associated with incidences of foodborne illness and food spoilage [14]. Food contamination emanates from the use of contaminated raw materials and ingredients in processing, poor personal hygiene, ineffective cleaning and sanitation of food contact surfaces, and contamination from food processing environment [13, 15–17].

Several microorganisms ranging from spoilage and pathogenic and indicator microorganisms are important in assessing safety, hygiene, and sanitary quality of foods and processing environments. These classes of microorganisms are comprised of total viable counts (TVC), yeasts and molds,* Staphylococcus aureus*, Enterobacteriaceae, coliforms, and* Escherichia coli*. TVC, yeasts, and molds are indicators of hygiene, sanitation, and microbial quality of both raw and processed foods. High total aerobic counts in foods are often associated with accelerated spoilage, hence deterioration in food quality [18]. High counts of* S. aureus* in foods and processing environment depict extensive handling and poor hand washing hygiene practices by food handlers. Consequentially,* S. aureus* counts above 10^5^ CFU/g produce heat-stable toxins responsible for staphylococcal food poisoning [19]. Coliforms,* E. coli*, and Enterobacteriaceae are useful indicators of water quality, personnel hygiene, and efficacy of cleaning and sanitation programs in food processing plants [20–22]. Control of contamination from persistent microorganisms in food processing environments can be achieved by application of quality assurance approaches such as GMPs and Environmental Monitoring Programs (EMPs). EMPs identify harborage niches for pathogens, spoilage, and indicator microorganisms that may act as a source of contamination and verifies adherence and implementation of GMPs in food processing environments [23].

Despite the economic and nutritional benefits accrued from OFSP puree processing, upholding food safety regulations is still a challenge that needs to be addressed. Like many small-scale food processing industries, OFSP puree processing is prone to microbial contamination attributed to low level of food safety knowledge and practices from food handlers [24] and poor hygiene within the processing environment [25]. Relatively high pH and high water activity in sweet potato puree further provide an excellent environment for growth of a wide array of both spoilage and pathogenic microorganisms [11]. There is lack of information on the level of compliance to GMPs and levels and sources of microbial contamination in OFSP puree processing in Kenya. There is need to generate data for identifying food safety risk areas and provide recommendations for improving hygiene, microbial quality, and safety of OFSP puree. The objective of the current study was therefore to determine the level of compliance to GMPs, sources, and levels of microbial contamination in OFSP puree processing plant in Homa Bay County, Kenya.

## 2. Material and Methods

### 2.1. Study Setting and Design

The study was conducted at a privately owned OFSP puree processing plant in Homa Bay County, Kenya. A cross-sectional analytical study design was used for data collection. Intensive observation and interviews guided by a structured GMPs checklist was used to assess the level of compliance to GMPs at the processing plant [25, 26]. The facility and its operations were evaluated for compliance to GMPs on aspects of suitability of buildings and sanitary facilities for food production, personal hygiene, food contact surfaces and equipment, pest control, and process control. The findings were categorized as either compliant or noncompliant, totaled, and converted into a percentage.

Samples for microbiological analysis were randomly collected from OFSP puree processing environment as described by Barros et al. [16]. A total of 62 samples comprising environmental samples, processing water, and OFSP samples were collected for microbial analysis. Swab samples from equipment, walls, floors, drains, and personnel hands were collected using 3M buffered swab sponges [27]. Sterile papers were used to outline areas of 30 cm^2^ and 60 cm^2^ on surfaces for swabbing. Samples from surfaces of equipment and installations were collected after cleaning. Samples from personnel hands were collected during working hours. OFSP samples were collected from three different batches at different processing stages: after washing, steaming, cooling, and cutting and packaging. All the samples were transported in a cold box filled with ice packs to the Department of Food Science, Nutrition and Technology, Upper Kabete Campus, University of Nairobi, and immediately analyzed for total aerobic counts, yeasts and molds, Enterobacteriaceae, coliforms,* Escherichia coli*, and* Staphylococcus aureus*.

### 2.2. Sample Preparation, Microbial Analysis, and Enumeration

All swab sample sponges (each presoaked in 10 ml buffer) were diluted with 90 ml sterile saline water (0.85% NaCl). The swab sponges were squeezed to release microbes from the surface before making successive serial dilutions. Twenty-five grams of process water and OFSP samples was each diluted with 225 ml of 0.85% NaCl before making subsequent serial dilutions as described by Gungor and Gokoglu [27].

#### 2.2.1. Determination of Total Viable Counts (TVC)

Total viable counts (TVC) were determined by spread plate method on Plate Count Agar (PCA, LAB, UK). The plates were incubated at 35°C for 48 hours as described by Pérez-Díaz et al. [11].

#### 2.2.2. Determination of Yeasts and Molds

Yeasts and molds were enumerated by plating 0.1 mL of each sample on Petri dishes with solidified Potato Dextrose Agar (PDA) (Oxoid, Hampshire). The plates were incubated at 25°C for 5 days as previously described by Gungor and Gokoglu [27].

#### 2.2.3. Determination of Enterobacteriaceae

Enterobacteriaceae group of microorganisms were determined by spread plating 0.1 mL of each sample on Violet Red Bile Glucose (VRBG) Agar (Oxoid, Hampshire, England). The plates were incubated at 37°C for 24 hours as described in a method by Hervert et al. [28].

#### 2.2.4. Detection of Coliforms and* Escherichia coli*

The presence of coliforms and* E. coli* was examined by plating 0.1 mL of each sample on Brilliance* E. coli*/coliform agar (Oxoid, Hampshire, England) according to the method by Sylvia et al. [29]. The plates were incubated at 37°C for 24 hours. Dark blue colonies were enumerated as* E. coli* while pink colonies were recorded as total coliforms.

#### 2.2.5. Detection of* Staphylococcus aureus*

*Staphylococcus aureus *was determined as per the method previously used by Gungor and Gokoglu [27]. Plating of 0.1 mL of each sample was done on Baird Parker agar (Oxoid, Hampshire, England). The plates were incubated at 37°C for 48 hours. Typical* S. aureus* colonies were enumerated and streaked in Brain Heart Infusion (BHI) broth (Oxoid) and further incubated at 37°C for 24 h. Typical* S. aureus* colonies were confirmed by coagulase test [30]. Test for coagulation was done by aseptically adding 0.1 mL of BHI culture to 0.3 mL of rabbit plasma in sterile hemolysis tubes. The tubes were incubated at 37°C and observed for coagulation after 6 hours.

#### 2.2.6. Enumeration of Microbial Colonies

Enumeration was done for plates with 30–300 colonies. All microbial counts were expressed as log_10_ CFU/g for OFSP samples, log_10_ CFU/ml for water sample, and log_10_ CFU/cm^2^ for swab samples.

### 2.3. Statistical Data Analysis

Compliance to GMPs was presented in tables. TVC, yeasts and molds,* S. aureus*, Enterobacteriaceae, coliforms, and* E. coli* counts were converted to log_10_ CFU units in Microsoft Excel (MS Office 365), exported to SPSS (IBM SPSS Version 20) for analysis before being tabulated as means and standard deviations. Analysis of variance and Tukey's test were used to determine statistical differences in the level of microbial contamination in the samples with a preset *P* value of 0.05.

## 3. Results and Discussion

### 3.1. Suitability and State of Hygiene of Equipment Used for OFSP Puree Processing

The suitability, design, cleaning, and sanitation of equipment for OFSP puree processing are shown in Table 1. The equipment surfaces were well designed for use in food processing but lack of sanitation procedures at the plant resulted in high microbial load of the surfaces as shown in Table 2. Lowest total aerobic counts were detected in packaging bags (6.6 ± 0.3 log cfu·cm^−2^) and the highest level of contamination was detected from weighing spoons (9.5 ± 0.0 log cfu·cm^−2^). The pureeing machine was least contaminated with yeasts and molds (4.3 ± 1.0 log cfu·cm^−2^). Low Enterobacteriaceae counts were detected in packaging bags and cooling trays with mean counts of 5.8 ± 0.6 and 5.8 ± 1.2 log cfu·cm^−2^, respectively. Highest counts of* S. aureus*, Enterobacteriaceae, and coliform counts with means 6.5 ± 0.0, 7.0 ± 0.0, and 6.7 ± 0.0 log cfu·cm^−2^, respectively, were detected on the inside cabin surface of the truck used in transportation of OFSP roots and OFSP puree. Highest yeasts and molds and* E. coli* counts with means 6.8 ± 0.5 and 5.3 ± 0.8 log cfu·cm^−2^, respectively, were obtained from buckets used for washing OFSP roots. The mean counts among different food equipment surfaces were statistically different (*P* < 0.05).

High counts above 10^5^ cfu·cm^−2^ for aerobic plate counts,* S. aureus*, yeasts and molds, coliforms, and Enterobacteriaceae were detected on more than 90% of all equipment surfaces indicating inadequate cleaning and sanitation procedures. This is similar to findings by Schlegelová et al. [22] that reported relatively high counts for total counts, enterococci,* E. coli*, and* Staphylococci *spp. on food equipment surfaces in dairy and meat processing plants. The high contamination level from equipment is attributed to lack of adherence to documented cleaning procedures by food handling personnel and lack of sanitation program at the establishment. Inefficient cleaning and sanitation of equipment surfaces lead to formation of biofilms, a potential source of food contamination [14, 22]. High counts on knives, cooling trays, tables, and pureeing machine were identified as primary sources of contamination in OFSP puree. Efficient cleaning and sanitation following documented procedures should always be done at the beginning of each work day, after every batch processing, and at the end of the day after processing to prevent formation of biofilms on equipment surfaces and contamination in OFSP puree processing.

### 3.2. Quality of Water Used in Orange Fleshed Sweet Potato Puree Processing

**Figure 1:**
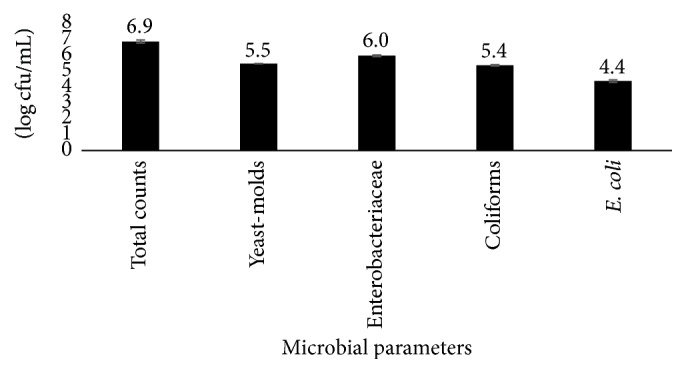
Microbial load in water for processing Orange Fleshed Sweet Potato puree; the values above the bars represent the mean ± SEM (standard error of the mean).

The level of microbial contamination in water for processing OFSP puree is shown in Figure 1. It was highly contaminated (>10^4^ CFU/ml) with Enterobacteriaceae, coliforms, and* Escherichia coli* due to nonexistence of water treatment (disinfection) program at the facility. As stipulated by Environmental Protection Agency [31], total coliforms and* E. coli* should be absent in 100 ml of water sample to be deemed suitable for drinking and food processing. It further recommends total counts not to exceed 500 cfu/ml. Use of untreated water from unknown sources contaminates equipment and foods prepared on these surfaces [32]. High Enterobacteriaceae, coliforms, and* E. coli* counts in water for OFSP puree processing indicated contamination with fecal matter, deterioration in water quality, and likelihood presence of enteric pathogens [32–34]. The water at the facility was therefore not suitable for use in processing. It was a possible source of contamination on equipment and personnel hands and consequently in OFSP puree. There was an urgent need to establish water disinfection program involving chlorination at the facility for preventing water to puree contamination.

### 3.3. Suitability of Buildings and State of Hygiene of Installations for OFSP Puree Processing

Prerequisite programs with respect to design of buildings, sanitary facilities, and pest control program in OFSP puree processing are shown in Tables 3, 4, and 5, respectively. Cleaning of walls, floors, and drains was not done efficiently as evidenced from our observation. This resulted in high level of contamination on installations as shown in Table 6. Total aerobic counts from floors, walls, and drains were identical with lowest counts (8.0 ± 0.9 log cfu·cm^−2^) recorded from floors and highest counts (8.7 ± 0.7 log cfu·cm^−2^) from drains. Yeasts and molds counts were above 10^5^ CFU·cm^−2^ but insignificant (*P* > 0.05) among the three installation points. Drains had the lowest contamination level for yeasts and molds (5.5 ± 1.3 log cfu·cm^−2^) while the walls had the highest counts (6.2 ± 0.6 log cfu·cm^−2^). The level of* Staphylococcus aureus* was low on floors (5.3 ± 0.4 log cfu·cm^−2^) and almost identical but high on walls (5.5 ± 0.0 log cfu·cm^−2^). Low and high Enterobacteriaceae counts were obtained from floors and drains with mean counts of 7.0 ± 0.4 and 7.2 ± 0.1 log cfu·cm^−2^, respectively. The level of contamination with coliforms was not significantly (*P* > 0.05) different. The lowest coliform counts were recorded from walls (6.4 ± 0.5 log cfu·cm^−2^) while highest counts were from drain surfaces (6.8 ± 0.4 log cfu·cm^−2^).* E. coli* counts from drains were significantly (*P* < 0.05) high as compared to floors and walls.

Floors, walls, and drains are high risk areas for bacterial growth and contamination in food processing plants [16]. Floors transfer contamination to food handlers' shoes who consequently circulate and disseminate the microorganisms within the establishment. Drains and floors offer a favorable environment for microbial growth if cleaning and sanitation are not done appropriately. High total counts, coliforms, and Enterobacteriaceae counts (>10^5^ log CFU·cm^−2^) from walls, floors, and drains in OFSP puree processing facility were attributed to inefficient cleaning of these areas. Similar results have been reported from meat processing environments in studies by Barros et al. and Ali et al. [16, 18]. Other than transferring contamination to trolleys and food handler's shoes, contaminated floors and walls in the facility can recontaminate personnel hands and equipment such as buckets, pallets, brushes, and cold boxes stored on the floor. Routine inspections, supervision of cleaning, and maintenance of installations and sanitary facilities can help in preventing proliferation and spread of microbial contamination within the OFSP puree facility.

### 3.4. Level of Personal Hygiene and Level of Microbial Load on OFSP Puree Handlers' Hands

**Figure 2:**
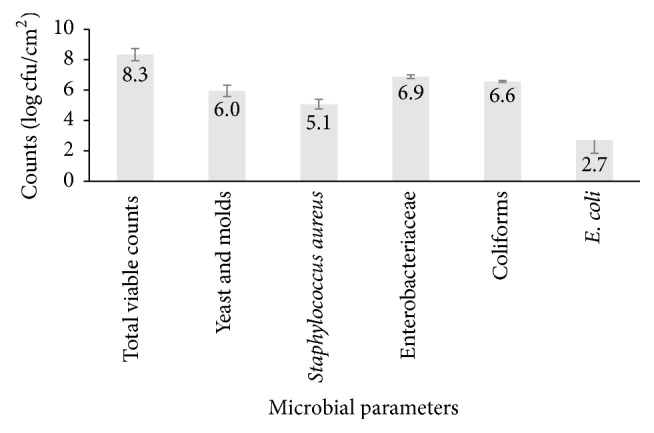
Microbial counts on Orange Fleshed Sweet Potato puree handlers' hands; the values above the bars represent the mean ± SEM (standard error of the mean).

Personal hygiene practices by OFSP puree handlers are shown in Table 7. Only 69% of the assessed practices on personal hygiene were considered compliant to food safety regulations for OFSP puree processing. The level of contamination on personnel's hands in OFSP puree processing in a decreasing order was 8.3 ± 0.6; 6.9 ± 0.4; 6.6 ± 0.2; 6.0 ± 1.0; 5.1 ± 0.9; and 2.7 ± 0.4 log cfu·cm^−2^ for total counts, Enterobacteriaceae, coliforms, yeasts and molds,* Staphylococcus aureus*, and* E. coli*, respectively (Figure 2).

High total counts and presence of potential pathogens (*S. aureus* and* E. coli*) on OFSP puree handlers' hands indicate low compliance to good hand washing hygiene practices by OFSP puree handlers and thus a potential source of contamination during processing of OFSP puree. Adherence to good personal hygiene by food handlers is important in preventing cross-contamination in food processing. The contact between food handlers and contaminated surfaces of equipment, phones, and walls classifies them as a potential source of contamination [35]. It is estimated that 10–20% of foodborne disease outbreaks occur as a result of contamination by food handlers [36]. All counts from personnel hands in this study were >10^5^ log CFU·cm^−2^ except for* E. coli*.* Staphylococcus aureus* was detected in all hand swab samples (100%) from OFSP puree handlers. This is contrary to findings by Al-Bahry et al. [37] that reported positive results for* S. aureus* in only 34% of asymptomatic food handlers in their study. From our observation, handling of OFSP in all the stages of processing was done with bare hands and more than 50% of all OFSP puree handlers were not regularly using soap during hand washing despite soap and hand washing instructions being supplied at every hand washing station. More than 50% of OFSP puree handlers failed to wash their hands after using their mobile phones during processing. Mobile phones have been reported as a source of bacterial and fungal contamination in food handling [35].

Provision of necessary food safety resources in a food production facility enhances food safety [38]. Gloves for handling OFSP puree and paper towels for hand drying were not provided at the OFSP puree processing facility. Gloves are crucial in preventing personnel's' hands to food contamination [39, 40]. It is documented that the transmission of bacteria is more likely to occur from wet hands than from dry hands [41]. This makes proper hand drying an essential component for effective hand hygiene in food processing facilities. Another study by Choi et al. [42] argues that provision of appropriate hand washing resources is not enough in enforcing proper hand hygiene. Results from their study indicated that 50% of food handlers in retail establishments failed to practice good hand washing hygiene despite being provided with necessary resources for hand hygiene. Training on personal hygiene, management support, and provision of food safety supplies such as gloves and paper towels should be considered in efforts to improve food safety in OFSP puree processing.

### 3.5. Process Control and Changes in Microbial Load in OFSP Puree during Processing

Compliance to quality control process parameters and changes in microbial load during processing of OFSP puree are shown in Tables 8 and 9, respectively. Only 44% of all quality control procedures were considered appropriate for OFSP puree processing. Total viable counts, yeasts and molds,* S. aureus*, Enterobacteriaceae, and coliforms counts were destroyed after steaming OFSP roots. Steaming is the main critical control point (CCP) for enhancing keeping quality and safety of OFSP puree. Several studies have reported cooking as an effective method in eliminating or reducing microorganisms in foods to safe levels [11, 27]. It is however a challenge destroying heat resistant spores and heat-stable toxins produced by pathogens such as* S. aureus* in a process involving mild heat treatment of foods.* S. aureus* counts in foods above 10^5^ CFU/g initiate production of heat-stable enterotoxins [37]. Staphylococcal food poisoning is one of the leading causes of foodborne illnesses worldwide caused by ingestion of food contaminated with preformed* S. aureus* enterotoxins [43]. Routine implementation of appropriate hygiene procedures and process control in OFSP puree processing can be an effective tool in reducing or eliminating contamination by pathogens or their toxins.

Microbial load significantly (*P* < 0.05) increased after cutting, cooling, pureeing, and packaging processes. High total counts and yeasts and mold counts in the puree indicated deterioration in keeping quality and hence accelerated spoilage. Haile et al. [44] reported lower TVC and yeasts and mold counts in porridge prepared from Orange Fleshed Sweet Potato (OFSP) contrary to the current findings. The high counts are attributed to contamination from equipment such as knives, cooling trays, pureeing machine, and packaging bags and contaminated water and poor personal hygiene by OFSP handlers. The presence of Enterobacteriaceae, coliforms, and* E. coli* in OFSP puree indicates fecal contamination and probable presence of enteropathogens due to cross-contamination from equipment, processing water, and food handling personnel. Additionally, high* S. aureus* counts detected in the puree was associated with contaminated equipment and poor hand washing hygiene practices from OFSP puree handlers. Contamination in OFSP puree can be eliminated by use of clean and sanitized equipment; clean and disinfected water; and adherence to good personal hygiene and appropriate handling practices by OFSP puree handlers.

### 3.6. Overall Level of Compliance to Good Manufacturing Practices in OFSP Puree Processing Plant

**Figure 3:**
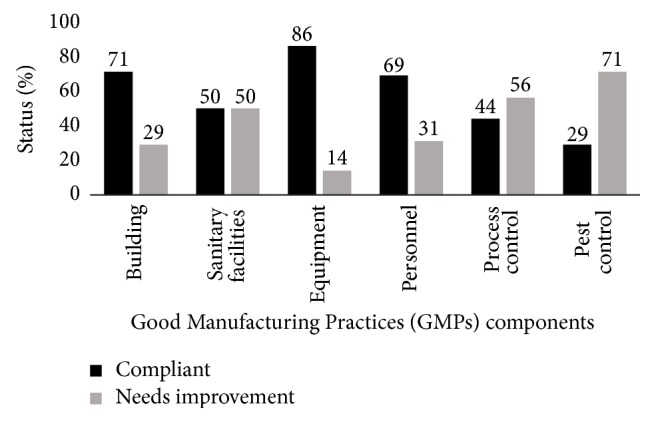
Overall level of compliance to GMPs in OFSP puree processing plant; the values above the bars represent the mean percentage score for compliance/noncompliance to GMPs.

The summarized levels of compliance to GMPs in puree processing with respect to buildings, sanitary facilities, personal hygiene, equipment, pest, and process control are shown in Figure 3.

The overall level of compliance to GMPs in puree processing was low with only 58% of all GMPs items in the present study being compliant. There is a need for urgent improvement on low scores for GMP items that covered areas on pest control, process control, sanitary facilities, personal hygiene, and suitability of the puree processing unit. Low compliance to GMPs is an impediment towards achieving food safety and quality standard requirements in OFSP puree processing. Kadariya et al. [43] emphasize strict adherence and implementation of GMPs as one of the best approaches for controlling microbial contamination in food processing. The Orange Fleshed Sweet Potato puree produced at the establishment was of unacceptable microbiological quality if it was to be commercialized as a ready-to-eat (RTE) food. The microbial load on equipment, food contact surfaces, personnel hands, processing water, and the processing environment was also above the recommended food safety legal limits. Even though there is limited data on safe microbial tolerable limits for food ingredients and food contact/preparation surfaces, some standards and regulations have been developed and adopted in some countries based on specifications provided by the International Commission for Microbiological Specification and other research studies. According to Food Standards Australia New Zealand [45], Centre for Food Safety [46], and Idris Ali and Immanuel [47], the levels considered acceptable/satisfactory in RTE foods are <10^4^ cfu for total viable counts, <10^2^ cfu for yeasts and molds, <20 cfu for* E. coli* and coliforms, <10^2^ cfu for Enterobacteriaceae, and <10^2^ cfu for* S. aureus*. The European Commission [48] previously suggested microbial level ranging from 0 to 10 cfu/cm^2^ on food equipment and food preparation surfaces and in processing environments as acceptable. Despite the existence of legal framework on food safety and quality in Kenya, the findings suggest that most small-scale food processors probably operate in disregard of food safety and quality controls [49, 50]. This is attributed to lack of qualified personnel, infrastructure, equipment, and other food safety resources necessary for hygienic processing, handling, storage, and distribution of food products [49, 50].

## 4. Conclusion

The present study revealed low compliance to GMPs in the only OFSP puree processing plant in Kenya. High microbial contamination levels on equipment, processing environment, and personnel hands revealed poor hygiene practices within the establishment. OFSP puree contamination emanated from equipment, processing water, and violation of food safety practices by puree handlers. It is therefore recommended to integrate Good Manufacturing Practices (GMPs), Good Hygiene Practices (GHPs), environmental monitoring programs, microbial risk assessments, and food safety trainings as quality control tools for enhancing food safety and quality of Orange Fleshed Sweet Potato puree.

## Figures and Tables

**Table 1:** Assessment of Good Manufacturing Practices for equipment used in OFSP puree processing.

Item	Yes	No	Status
(1) Are all cleaned food equipment surfaces sanitized as necessary to prevent contamination of the product?		√	Noncompliant
(2) Is the equipment designed or otherwise suitable for use in OFSP puree processing?	√		Compliant
(3) Is there a build-up of food or other material on the equipment?		√	Compliant
(4) Is there any build-up or seepage of cleaning solvents or lubricants on your equipment which can contaminate food?		√	Compliant
(5) Is the equipment hard to dissemble for clean-up and inspection?		√	Compliant
(6) Is there a lot of dead space in and around the machinery where the food and other debris can collect and act as nest for insects and bacteria?		√	Compliant
(7) Can the surface of the equipment be sanitized?	√		Compliant

**Table 2:** Microbial Counts on equipment surfaces in OFSP puree processing plant^1^ (log Mean CFU·cm^−2^).

Sample	TVC	Yeast-molds	*S. aureus*	Enterobacteriaceae	Coliforms	*E. coli*
OFSP buckets	7.71 ± 0.24^bcde^	6.79 ± 0.45^ef^	5.08 ± 0.32^bcd^	7.33 ± 0.09^bcd^	6.51 ± 0.10^efgh^	5.29 ± 0.75^b^
OFSP scrub brushes	7.83 ± 0.02^bcde^	6.32 ± 0.01^def^	4.52 ± 0.21^bcd^	7.28 ± 0.03^bcd^	6.44 ± 0.13^efgh^	4.34 ± 0.15^b^
Knives	6.57 ± 0.32^bc^	4.35 ± 0.29^bcde^	5.06 ± 0.13^bcd^	6.24 ± 0.02^bcd^	5.51 ± 0.15^def^	4.79 ± 0.33^b^
Tables	8.12 ± 1.24^bcde^	5.73 ± 1.13^cdef^	4.44 ± 0.84^bcd^	6.71 ± 0.07^bcd^	6.35 ± 0.54^defgh^	2.16 ± 3.74^a^
Cooling trays	7.45 ± 1.37^bcde^	4.74 ± 0.27^cdef^	4.68 ± 0.55^bcd^	5.83 ± 1.20^bcd^	5.45 ± 1.16^cde^	2.68 ± 2.10^a^
Puree machine	7.63 ± 0.81^bcde^	4.29 ± 1.03^bcd^	4.43 ± 0.63^bcd^	6.34 ± 0.45^bcd^	5.58 ± 0.20^defg^	2.04 ± 2.23^a^
Weighing spoons	9.47 ± 0.03^e^	6.45 ± 0.08^def^	5.41 ± 0.14^cd^	6.43 ± 0.10^bc^	6.17 ± 0.12^defgh^	1.81 ± 2.57^a^
Packaging bags	6.28 ± 0.09^bc^	4.83 ± 1.35^cdef^	3.16 ± 0.43^bc^	5.83 ± 0.64^bc^	5.65 ± 0.73^defgh^	nd^*∗*^
Packaging machine	9.10 ± 0.74^de^	6.15 ± 0.51^cde^	5.15 ± 0.04^bcde^	6.77 ± 0.05^cde^	6.45 ± 0.02^defg^	3.58 ± 0.08^b^
Freezers	7.46 ± 0.13^bcde^	5.95 ± 0.83^cdef^	4.89 ± 1.53^bcd^	6.64 ± 0.11^bcd^	6.32 ± 0.40^defgh^	1.21 ± 2.11^b^
Cold boxes	7.97 ± 0.13^bcde^	5.48 ± 0.55^cdef^	5.65 ± 0.11^cd^	6.27 ± 0.17^cd^	5.58 ± 0.24^defg^	4.36 ± 0.76^b^
Shipment vehicle	8.33 ± 0.01^bcde^	5.28 ± 0.00^cde^	6.45 ± 0.02^e^	7.43 ± 0.03^e^	6.68 ± 0.01^fgh^	4.70 ± 0.96^b^

^1^All values reflect mean counts and standard deviation. Values bearing different superscript letters in each column are significantly different (*P* < 0.05); TVC: total viable counts; nd^*∗*^: microbial parameter not detected.

**Table 3:** Assessment of GMPs for buildings, grounds, and structures for OFSP puree processing.

Item	Yes	No	Status
(1) Is the OFSP puree processing plant located in an environment free of dust?	√		Compliant
(2) Is the area around the plant clear of litter, weeds, grass and brush?	√		Compliant
(3) Is there any standing water on the ground which might also attract pests?		√	Compliant
(4) Are floors, walls and drains properly cleaned?		√	Noncompliant
(5) Are floors made of alkali and acid resistant material?	√		Compliant
(6) Do floors have sufficient slope to avoid water stagnation?		√	Compliant
(7) Do production area doors and windows to the outside have fine mesh screens to keep out insects? If not are they tightly sealed?	√		Compliant
(8) Have all holes and cracks been filled so as not provide hiding places or entry points for pests?	√		Compliant
(9) Are there any presence of domestic animals such as cats and dogs?		√	Compliant
(10) Are the hand washing facilities furnishedwith soap and paper towels?		√	Noncompliant
(11) Are there any leaks in the roof, sky lights, windows or overhead piping?		√	Compliant
(12) Are drains adequately designed to handle the volume of waste water?		√	Noncompliant
(13) Are all drains fitted with screens and waste traps to prevent pest entry into the processing areas?	√		Compliant
(14) Are the overhead lights covered with shields to prevent contamination of products by broken glass in case the lamps burst?		√	Noncompliant

**Table 4:** Good Manufacturing Practices assessment for sanitary facilities in puree processing.

Item	Yes	No	Status
(1) Is trash, debris and clutter picked up, both inside and outside, so as not to provide hiding places for pests?	√		Compliant
(2) Are OFSP puree handlers provided with designated areas for eating, drinking and using tobacco products?		√	Noncompliant
(3) Is food spilled cleaned up quickly so as not to attract pests or breed bacteria?	√		Compliant
(4) Is garbage quickly removed and dumped in appropriate bins?	√		Compliant
(5) Is the garbage kept covered?		√	Noncompliant
(6) Is the water used in the plant treated?		√	Noncompliant

**Table 5:** Assessment of GMPs on pest control programs for OFSP puree processing.

Item	Yes	No	Comment
(1) Do you have professional pest control services?		√	Noncompliant
(2) Do you have documentation on what chemicals are being used?		√	Noncompliant
(3) Are mites, weevils or other insects apparent in the plant?	√		Noncompliant
(4) Do you have enough bait stations?	√		Compliant
(5) Are safety rules observed during fumigation?	√		Compliant
(6) Are the pest control logs and documentation readily available?		√	Noncompliant
(7) Are pesticides or application equipment readily available?		√	Noncompliant

**Table 6:** Microbial counts of installations in OFSP puree processing plant^1^ (log Mean CFU·cm^−2^).

Sample	TVC	Yeast-Molds	*S. aureus*	Enterobacteriaceae	Coliforms	*E. coli*
Floors	8.03 ± 0.87^bcde^	5.71 ± 0.94^cdef^	5.31 ± 0.40^cd^	6.98 ± 0.35^cd^	6.66 ± 0.22^gh^	2.75 ± 3.20^a^
Walls	8.69 ± 0.65^bcde^	6.17 ± 0.56^cdef^	5.54 ± 0.03^cd^	7.00 ± 0.26^cd^	6.43 ± 0.54^defgh^	1.45 ± 2.51^a^
Drains	8.71 ± 0.73^bcde^	5.46 ± 1.29^cdef^	5.33 ± 0.90^cd^	7.20 ± 0.06^cd^	6.80 ± 0.36^h^	3.50 ± 3.14^b^

^1^All values reflect mean counts and standard deviation. Values bearing different superscript letters in each column are significantly different (*P* < 0.05); TVC: total viable counts.

**Table 7:** Assessment of personal hygiene practices in OFSP puree processing.

Item	Yes	No	Status
(1) Are there instructions on how to be suitably dressed to enter production areas?	√		Compliant
(2) Do food handlers wash their hands in clean water before handling and preparation of food?	√		Compliant
(3) Do operators wash their hands each time after visiting the toilet?	√		Compliant
(4) Are employees provided paper towels and hand sanitizers?		√	Noncompliant
(5) Are operator's clothes clean and presentable?	√		Compliant
(6) Are the food handlers observed to have any illnesses, infections, or injuries (boils, cuts) that can contaminate food in the production area?		√	Compliant
(7) Do food handlers use protective clothing when handling and preparing food?	√		Compliant
(8) Do food handlers handle food with bare hands?	√		Noncompliant
(9) Are disposable or reusable gloves provided?		√	Noncompliant
(10) Do operators have clean short nails?	√		Compliant
(11) Is their hair covered when handling and processing food?	√		Compliant
(12) Do food handlers use mobile phones while handling and preparing food?	√		Noncomplaint
(13) Do food handlers wear jewelry, rings, watches, fingernail polish or bandages in the processing establishment?		√	Compliant
(14) Do food handlers smoke/chew while handling and preparing food?		√	Compliant
(15) Do operators use the same equipment and surfaces in preparing raw and processed food?		√	Compliant
(16) Do food handlers blow their nose/cough while handling and preparing food?		√	Compliant

**Table 8:** Good Manufacturing Practices for process control in OFSP puree processing.

Item	Yes	No	Status
(1) Are OFSP roots and puree stored on a first in, first out basis to reduce the possibility of contamination through spoilage?	√		Compliant
(2) Is a thermometer for recording heating temperature for OFSP roots provided?		√	Noncompliant
(3) Is OFSP puree dated to ensure a proper rotation and for internal tracking purposes		√	Noncompliant
(4) Are raw materials and the puree overstocked?		√	Compliant
(5) Are trucks inspected, cleaned and sanitized?		√	Noncompliant
(6) Are incoming OFSP roots inspected for damage or contamination so that they can be rejected?	√		Compliant
(7) Are freezer temperatures monitored and recorded?		√	Noncompliant
(8) Are food related items stored together with non-food related items?		√	Compliant
(9) Do you have an effective recall procedure set up?		√	Noncompliant

**Table 9:** Microbial counts of OFSP at different stages of processing in OFSP puree processing plant^1^ (log Mean CFU/g).

Processing stages	TVC	Yeasts-molds	*S. aureus*	Enterobacteriaceae	Coliforms	*E. coli*
Raw OFSP	7.19 ± 0.27^bcd^	2.51 ± 0.30^b^	3.01 ± 0.15^b^	4.57 ± 0.20^b^	3.74 ± 0.19^b^	nd^*∗*^
Steaming	nd^*∗*^	nd^*∗*^	nd^*∗*^	nd^*∗*^	nd^*∗*^	nd^*∗*^
Cooling and Slicing	7.12 ± 0.21^bcd^	nd^*∗*^	2.93 ± 2.54^b^	4.62 ± 0.15^b^	4.37 ± 0.13^bc^	nd^*∗*^
Packaging	7.96 ± 0.57^bcde^	4.01 ± 0.33^bc^	5.88 ± 0.53^cd^	6.55 ± 0.18^cd^	5.82 ± 0.13^defgh^	4.77 ± 0.45^b^

^1^All values reflect mean counts and standard deviation. Values bearing different superscript letters in each column are significantly different (*P* < 0.05); TVC: total viable counts; nd^*∗*^: not detected.
